# Supplementary material for: DMRforPairs: identifying Differentially Methylated Regions between unique samples using array based methylation profiles
Source: BMC Bioinformatics. 2014 May 15;15:141. doi: 10.1186/1471-2105-15-141 (PMC4046028; doi:10.1186/1471-2105-15-141)
Supplement: Additional file 2 — DMRforPairs output for the comparison of A431-MCF7 and NA17018-NA17105. Please start from the HTML files in each folder. Available via the BMC Bioinformatics website. [file 1471-2105-15-141-S2.zip › 1394847754114233_MOESM2_ESM/A431_MCF7/figures/15292.pdf]

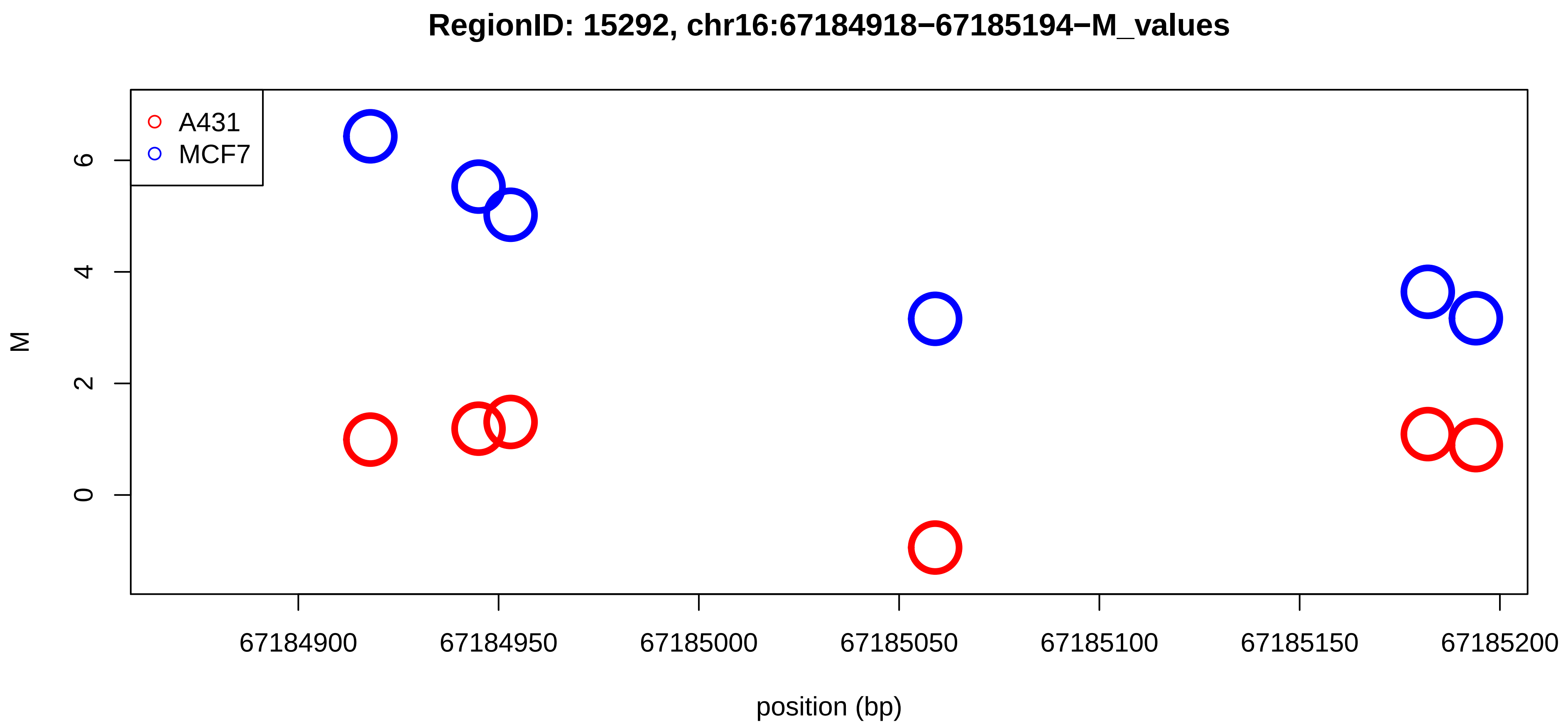

RegionID: 15292, chr16:67184918−67185194−M_values
A431
MCF7
M
0
2
4
6
67184900
67184950
67185000
67185050
67185100
67185150
67185200
position (bp)

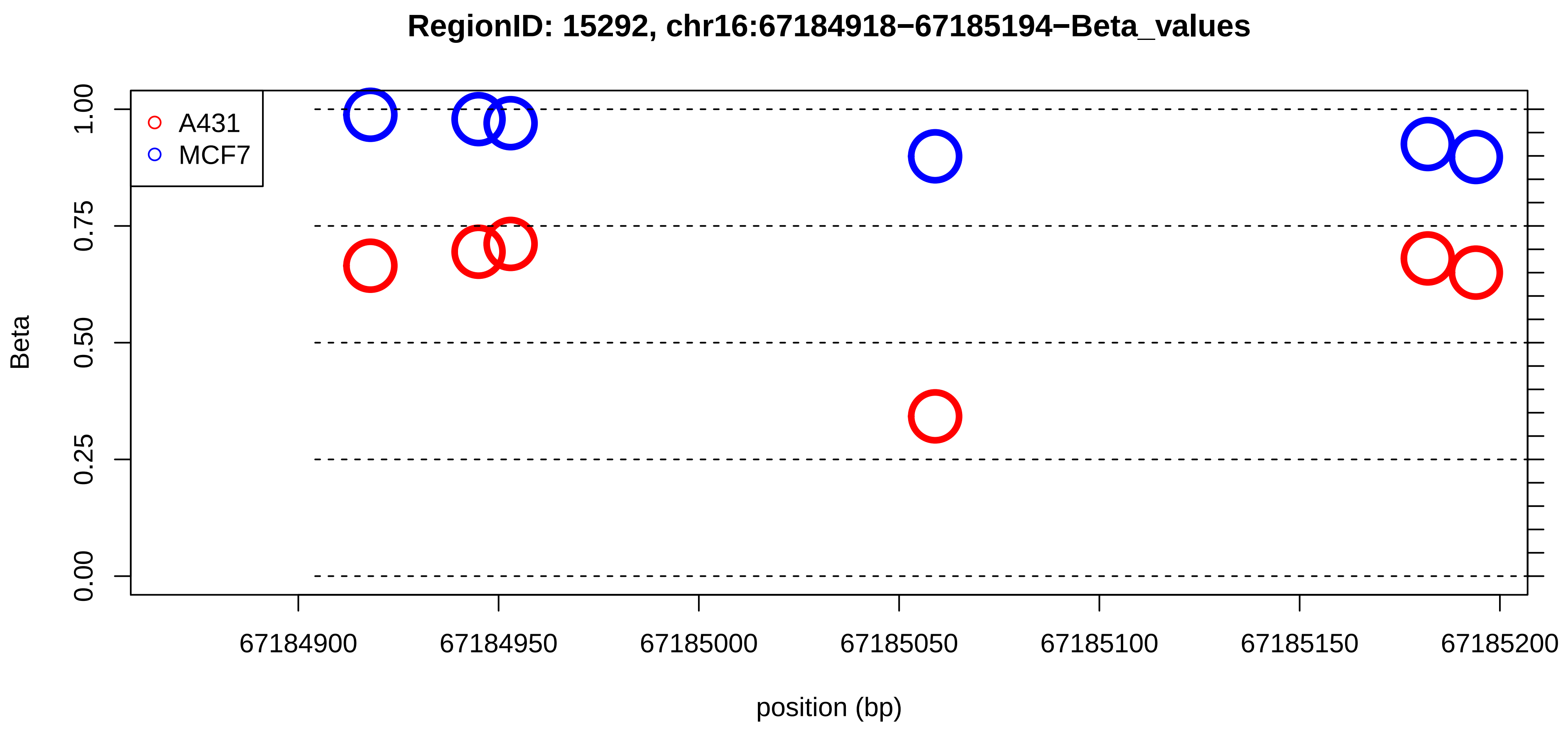

RegionID: 15292, chr16:67184918−67185194−Beta_values
A431
MCF7
Beta
0.00
0.25
0.50
0.75
1.00
67184900
67184950
67185000
67185050
67185100
67185150
67185200
position (bp)

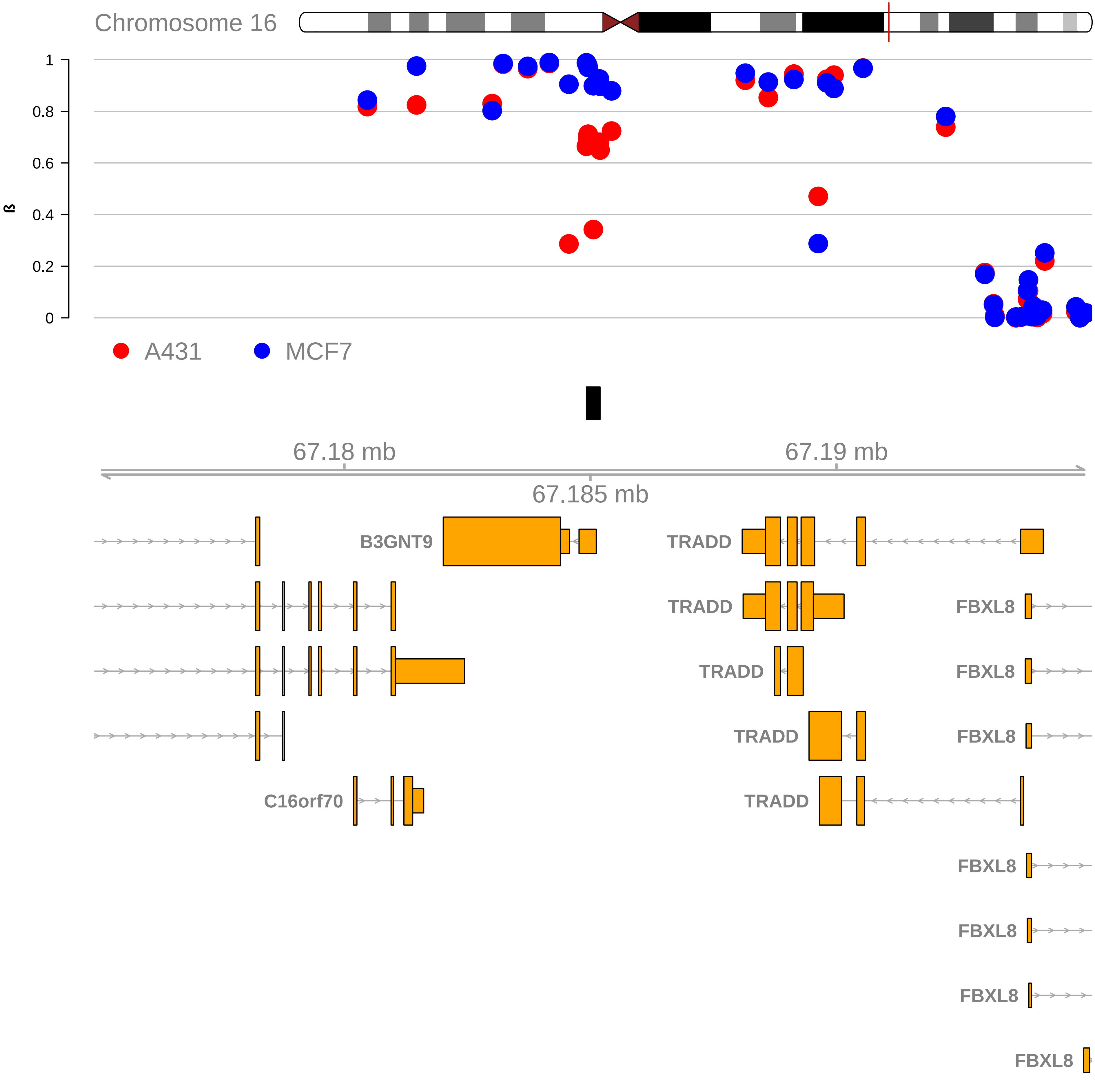

Chromosome 16
1
0.8
0.6
0.4
0.2
0
β
A431
MCF7
67.18 mb
67.19 mb
67.185 mb
B3GNT9
TRADD
FBXL8
C16orf70
